# SponDT (Spondylodiscitis Diagnosis and Treatment): spondylodiscitis scoring system

**DOI:** 10.1186/s13018-019-1134-9

**Published:** 2019-04-11

**Authors:** Lars Homagk, Daniel Marmelstein, Nadine Homagk, Gunther O. Hofmann

**Affiliations:** 1 Praxisklinik Dr. Homagk – MVZ GmbH, 06667 Weißenfels, Germany; 2 Centre for Spinal Cord Injuries and Department of Orthopedics, BG Kliniken Bergmannstrost, 06112 Halle (Saale), Germany; 3 Clinic of Trauma Hand- und Reconstructive Surgery, Friedrich-Schiller-University Jena, Jena, Germany; 4 Praxisklinik Dr. Homagk, Markt 3, 06618 Naumburg, Germany

**Keywords:** Spondylodiscitis, Scoring system, Classification of severity

## Abstract

**Background:**

Spondylodiscitis is a chameleon among infectious diseases due to the lack of specific symptoms with which it is associated. It is nevertheless a serious infection, with 7% mortality of hospitalized patients, in large part because of delayed diagnosis. The aim of this study was to develop a diagnosis and course-of-disease index to optimize its treatment.

**Material and methods:**

Through analysis of 296 patients between January 1998 and December 2013, we developed a scoring system for spondylodiscitis, which we term SponDT (Spondylodiscitis Diagnosis and Treatment) based on three traits: (1) the inflammatory marker C-reactive protein (CRP) (mg/dl), (2) pain according to a numeric rating scale (NRS) and (3) magnetic resonance imaging (MRI), to monitor its progression following treatment.

**Results:**

The number of patients receiving treatment increased over the past 15 years of our study. We also found an increasing age of patients at the point of diagnosis across the study, with an average age of 67.7 years. In 34% of patients, spondylodiscitis developed spontaneously. Almost 70% of them did not receive treatment until the first diagnosis using SponDT. Following treatment against spondylodiscitis, pain intensity decreased from 6.0 to 3.1 NRS. The inflammatory markers also decreased (CRP from 119.2 to 46.7 mg/dl). Similarly, MRI revealed a regression in inflammation following treatment. By employing SponDT, patients were diagnosed and entered into treatment with a score of 5.6 (severe spondylodiscitis) and discharged with a score of 2.4 (light/healed spondylodiscitis).

**Conclusion:**

SponDT can be used to support the diagnosis of spondylodiscitis, particularly in patients suffering from back pain and elevated levels of inflammation, and can be used during the course of treatment to optimize control of therapy.

**Level of evidence:**

IIa—evidence from at least one well-designed controlled trial which is not randomized

## Introduction

Spondylodiscitis is an infectious inflammation that affects the vertebrae, vertebral discs and adjacent structures. It may have a bacterial or non-bacterial aetiology, and, in the former case, a relatively broad spectrum of pathogens is considered to induce spondylodiscitis; the largest proportion of spondylodiscitis cases is caused by *Staphylococcus aureus* [1–3]. The incidence of spondylodiscitis is on average seven per million, with men three times more frequently affected than women. Although spondylodiscitis is a rare disease, the widespread use of drugs that suppress the immune system and an ageing population have led to an increase in cases of infection [3, 4]. Though the disease is most often seen in the sixth decade of life, it can occur at any age. In addition to age, risk factors include diabetes mellitus, malnutrition and disorders inducing a loss of weight, steroid therapy, rheumatic diseases and spinal surgery. Often, the actual source of infection is no longer detectable at diagnosis [5–7].

Before the use of antibiotics, spondylodiscitis thought to have led to mortality of 25–71% of cases. The rate of mortality lies at 2–12% [2, 3, 8]. Pathogenesis is through blood-borne transport of pathogens following surgical intervention or following growth of a soft tissue infection. Spontaneous spondylodiscitis also occurs via a haematogenous route [9–11]. The number of cases of spondylodiscitis arising from a post-operative infection is less than those arising from blood-borne infections. The most common site of spondylodiscitis is the lumbar spine (60%), followed by the breast (30%) and the cervical spine (10%) [3, 5, 7].

Spondylodiscitis can characterize in its early stages by rather nonspecific clinical symptoms such as mild fever, general malaise, weakness and weight loss. Because of the lack of specificity of the symptoms, there is often a delay of several weeks and up to 6 months in its diagnosis [2, 3, 9, 12]. The disease can also be associated with acute sepsis, multiorgan failure and neurological symptoms. These may be due to a para- or intraspinal abscess or destruction of bony parts of vertebrae. The mortality of acute spondylodiscitis cases can rise to 17% [2, 3].

In addition to neurological examination in the clinic, early diagnostic parameters of spondylodiscitis include elevated body temperature, increased C-reactive protein (CRP) titres and an increased white blood cell count. A serious limitation in diagnosis is that these parameters, though having high specificity for the detection of an infection, have only low sensitivity for the detection of spondylodiscitis [13, 14]. Therefore, a further prerequisite for adequate treatment of spondylodiscitis beyond early detection is an accurate diagnosis, which can often only be determined by histological analysis or direct detection of causative pathogens. One route of pathogen detection is via culturing of blood or the cultivation of pathogens from biopsy material [1, 15, 16].

Culturing of blood is the easiest, cheapest and most effective method for confirming the presence of a pathogen. Since haematogenous spondylodiscitis is usually caused by a single pathogen [17], there is a high probability of detecting it via culturing of blood, for which both aerobic and anaerobic blood cultures should be employed. Pathogens could be detected in 40–70% of patients who had not been treated with antibiotics [15, 18]. Up to 82% of spondylodiscitis cases with an epidural abscess have pathogens detectable in the blood [19].

X-ray images of spinal segments affected by spondylodiscitis occasionally exhibit primary non-specific changes associated with osteolysis and shadowing of the paravertebral soft tissue, suggestive of a spinal abscess. MRI is more sensitive and specific for the diagnosis of spondylodiscitis and therefore the method of choice [11, 20]. If MRI does not allow diagnosis when spondylodiscitis is suspected, it is then important to undertake a computer tomography (CT) in conjunction with a contrast medium (edge enhancement of the paravertebral abscesses) for the assessment of the stability of bony structures. Scintigraphy is of low diagnostic specificity and sensitivity but might help in following the course of infection. Image intensification or CT scan-based puncturing for pathogen detection can be performed transpedicular or extrapedicular but is only successful in about 50% of the cases; it is only recommended where conservative therapy is sought [11, 12].

Spinal cord or nerve root compression with abscesses, destructive lesions that lead to bony spinal instability, and other abscesses should be treated surgically [8, 21]. The aim of the surgery is to achieve a radical and complete elimination of any inflamed or necrotic tissue, to clean the site of infection and to restore the stability of the spine. A variety of materials can be used to stabilize the spine, depending on the size of the damaged area to be cleaned, e.g. mono- or bicortical pelvic chips, fibulainterponate or, now more rarely, cortico-tibial chips as well as implants such as titanium cages [3, 22]. Increasingly, a minimally invasive surgical procedure is advised [5, 21, 22].

Considering the difficulty in diagnosis of the disease, the aim of this study was to develop a tool to control the treatment of spondylodiscitis.

## Material and methods

In this retrospective study, we took into consideration all cases of spondylodiscitis (total 296 patients) from January 1998 through to December 2013. We collected their medical history, associated pathogens, location of spondylodiscitis, time spent in hospital, number of operations, age, pre-admittance treatment, possible causes of illness and non-radiological diagnosis (clinical, laboratory, microbiology). We assessed their C-reactive protein (CRP) levels in addition to the usual routine chemical laboratory diagnostics: blood count, coagulation, electrolytes, liver enzymes, kidney enzymes and urine. To confirm the diagnosis of spondylodiscitis and exclude other causes, we evaluated original radiographs; computed tomography images, possibly with aspiration (CT); and magnetic resonance imaging (MRI).

From the in-house electronic medical records, we generated a measure of the pain intensity of patients using a numerical rating scale (NRS), which ranged from 0 (lowest pain intensity) to 10 (highest pain intensity). The NRS was based on interviews with patients and additional daily recording of pain intensity, as expressed by the patient at rest, though changes in the treatment for pain and through patient mobility [23].

As part of the pre-operative evaluation, the general condition of the patient by the anaesthetist was recorded using the American Society of Asesthesiologists (ASA) classification. This scheme, proposed in 1940 by the American Society of Anesthesiologists, is widely used in medicine for classifying patients into groups with respect to their physical condition. It distinguishes patients before anaesthesia on the basis of their systemic illnesses [24, 25].

## Results and discussion

**Fig. 1:**
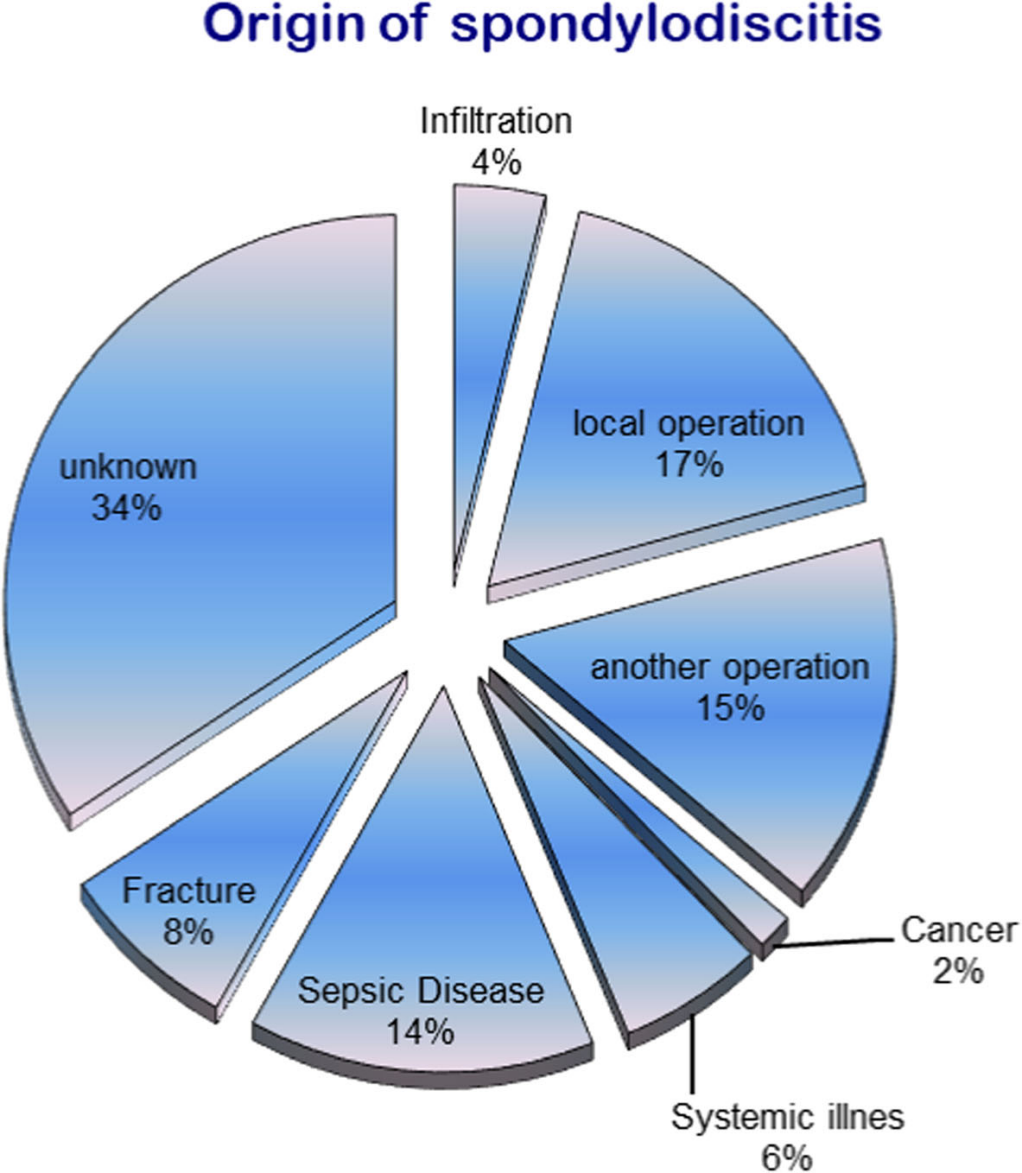
Origin of spondylodiscitis in a total of 296 patients

The 296 patients included in our study had a mean age of 67.3 years. 66.2% were 60 to 80 years old and 56.7% were male. In 34.3% of patients, the cause of spondylodiscitis could not be determined. The most common known causes were medical interventional measures (e.g. infiltration, a spine operation or another operation; 36.2%), followed by previous sepsis (14.3%) or vertebral fracture (7.5%). Figure 1 gives other causes of spondylodiscitis.

**Fig. 2:**
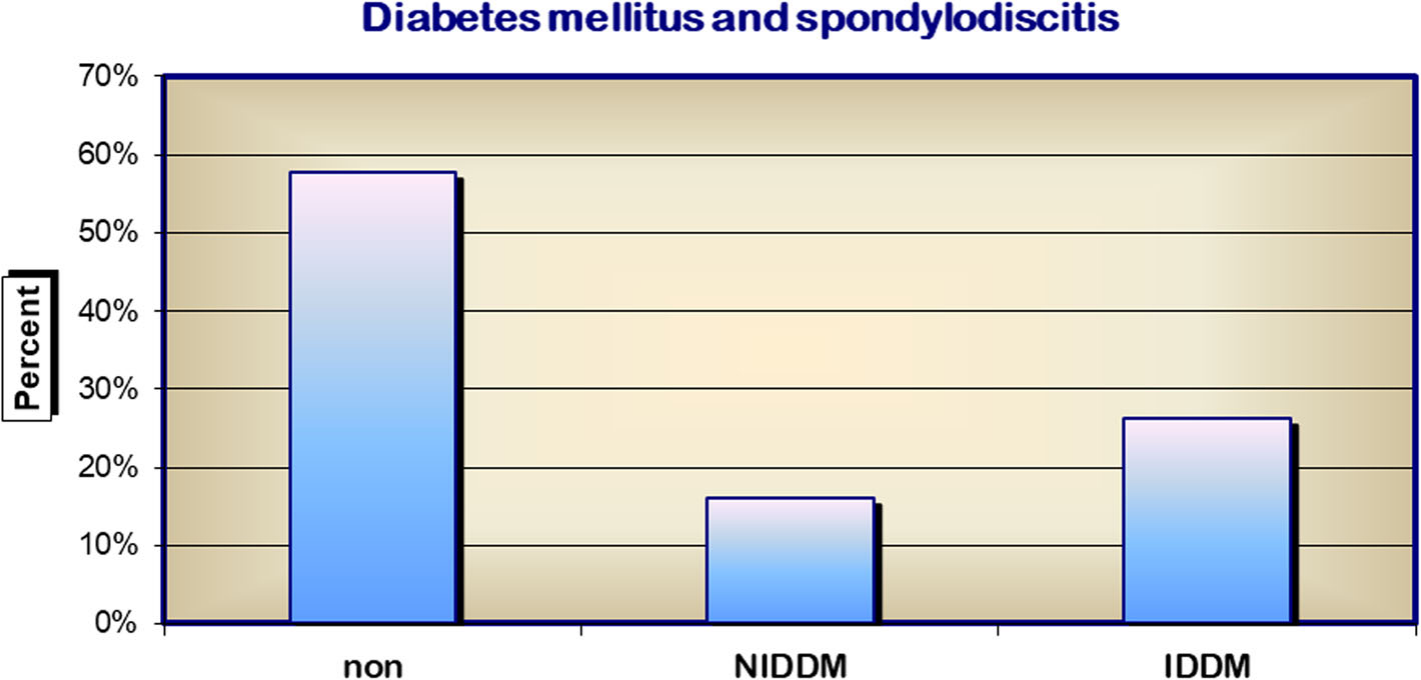
Diabetes mellitus and spondylodiscitis. NIDDM non-insulin dependent diabetes mellitus, IDDM insulin dependent diabetes mellitus

ASA classification placed > 66% of patients in stages III and IV at first diagnosis. There was no significant change over the course of the study. Diabetes mellitus was recorded in a total of 42.2% of patients, 26.2% of whom were already receiving treatment with insulin (Fig. 2).

**Fig. 3:**
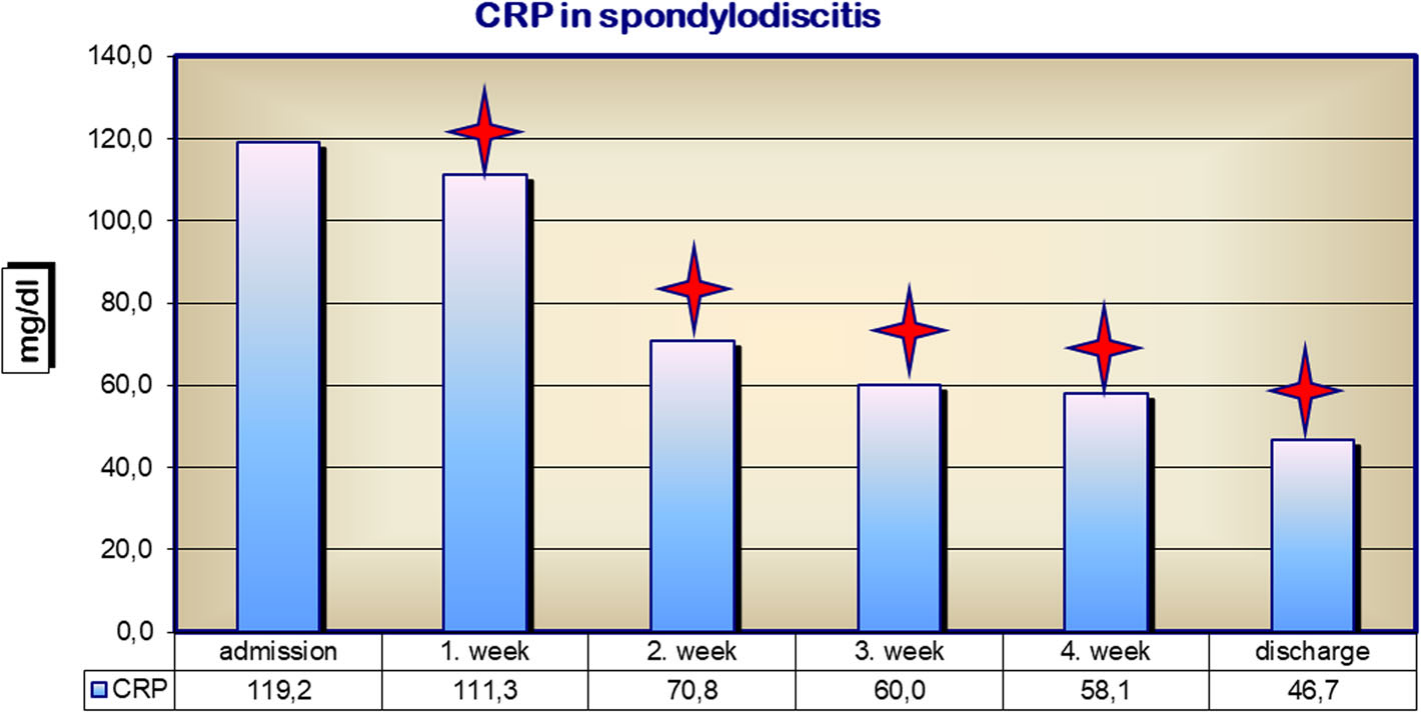
C-reactive protein (milligrammes per decilitre) on admission and during course of treatment; *p < 0.05

Patients were generally in a reduced state of health when first diagnosed with spondylodiscitis, which is reflected in their poor routine chemical laboratory diagnostics. In addition, their C-reactive protein (CRP) level was 119.2 mg/dl, well above the standard value of < 10 mg/dl, though it fell to 46.7 mg/dl following surgery and antibiotic treatment (Fig. 3).

**Fig. 4:**
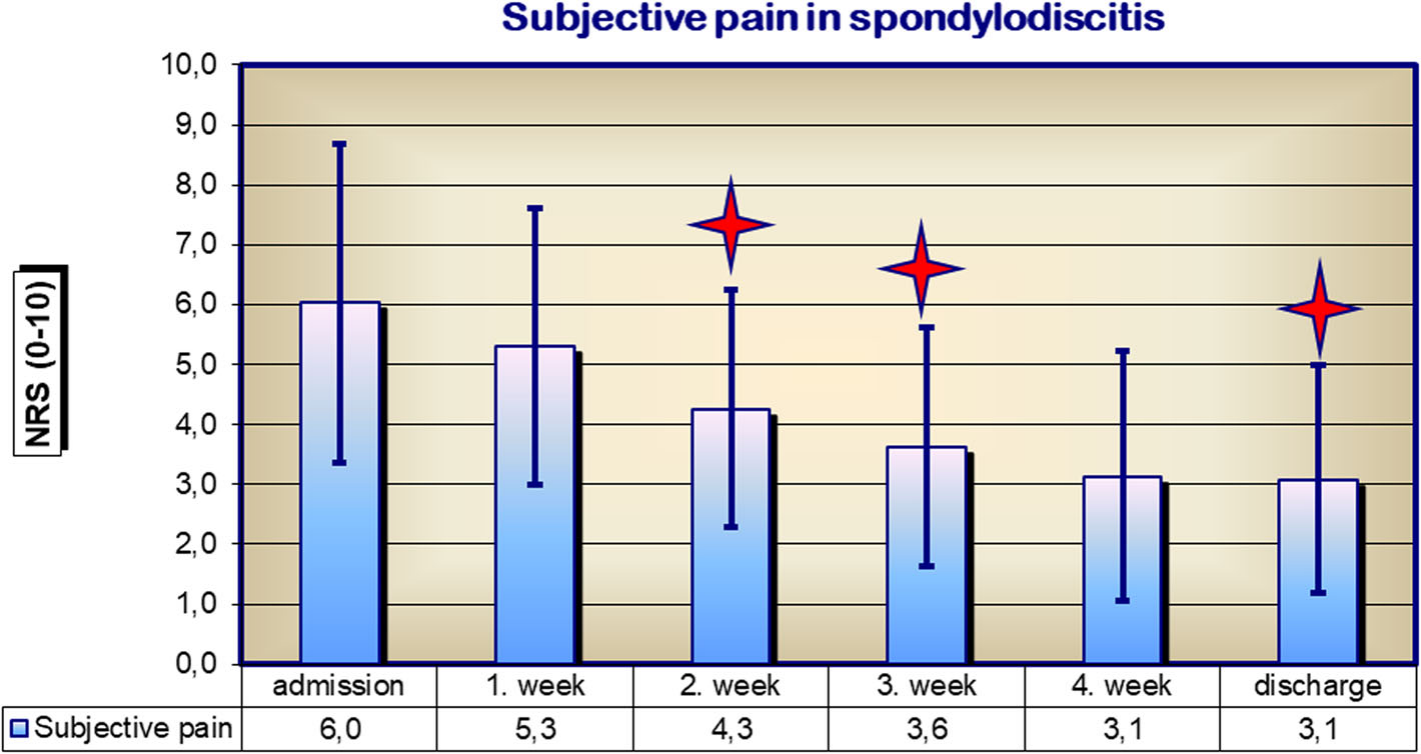
Subjective perception of pain (NRS) of spondylodiscitis patients on admission and during the course of treatment; *p < 0.05

Leukocyte levels of spondylodiscitis patients at first diagnosis were 10.6 Gpt/l, not significantly higher than the normal value of 9.5 Gpt/l. They generally fell across the course of treatment for spondylodiscitis to the normal value of 9.5 on discharge, though they exhibited a transient rise at week 4 after initiation of treatment. The subjective perception of patient pain decreased significantly from 6.0 NRS to 3.1 NRS following diagnosis and treatment for spondylodiscitis (Fig. 4).

On admission, all 296 patients received imaging, of which 64% were conventionally x-rayed and 78% involved CT examination. MRI was performed on 74% of all patients. With the introduction of SponDT in our daily procedure in 2004, every patient received MR imaging. Only exceptions are a pacemaker or really bad condition of patients. This MRI was followed by morphological classification of patients, following Flamme et al. [20] (see Table 1).

**Table 1:** MR-based morphological classification staging of spondylodiscitis

Stage	0	1	2	3
MRI	None	Spondylitis or discitis (T2 intensity, contrast enhancement)	Spondylodiscitis (T1 intensity, T2, KM intensity, contrast enhancement in BS/WK, structural disorders)	Spondylodiscitis with a paravertebral abscess

**Fig. 5:**
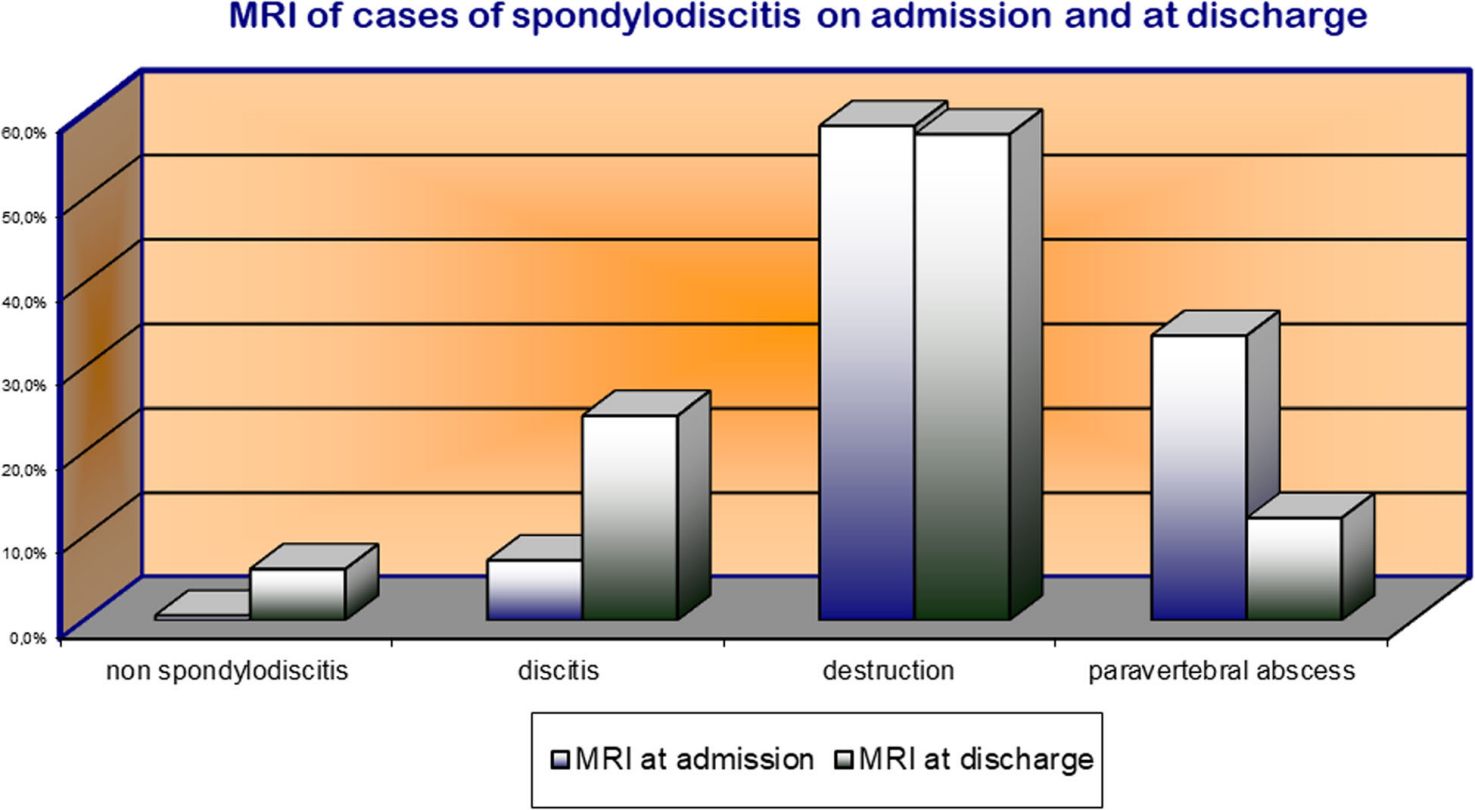
MRI of cases of spondylodiscitis on admission and at discharge

In MRI follow-up imaging, it was found that spondylodiscitis could not be detected in 6.1% of patients studied. Paravertebral abscesses were also significantly reduced in prevalence from an initial 33.7% of patients to 12.1% of patients (Fig. 5).

Based on these data, we developed a spondylodiscitis diagnosis and treatment score, in short SponDT (Table 2), to be used as a treatment monitoring tool.

**Table 2:** Spondylodiscitis scoring system for diagnosis and therapy control using SponDT

Score value	0	1	2	3
CRP (mg/dl)	< 10	11–50	51–150	> 150
Pain (NRS)	< 3	3–5	6 – 8	> 8
MRI	None/	Discitis	Spondylodiscitis	Spondylodiscitis with abcesses
	Res			

According to the score value, following the grade of severity could be performed (Table 3):

**Table 3:** Classification of the severity of spondylodiscitis using SponDT

Severity	Point score using SponDT
Severe spondylodiscitis	Over 6
Moderate spondylodiscitis	From 3 to 5
Light/healed spondylodiscitis	Under 3

**Fig. 6:**
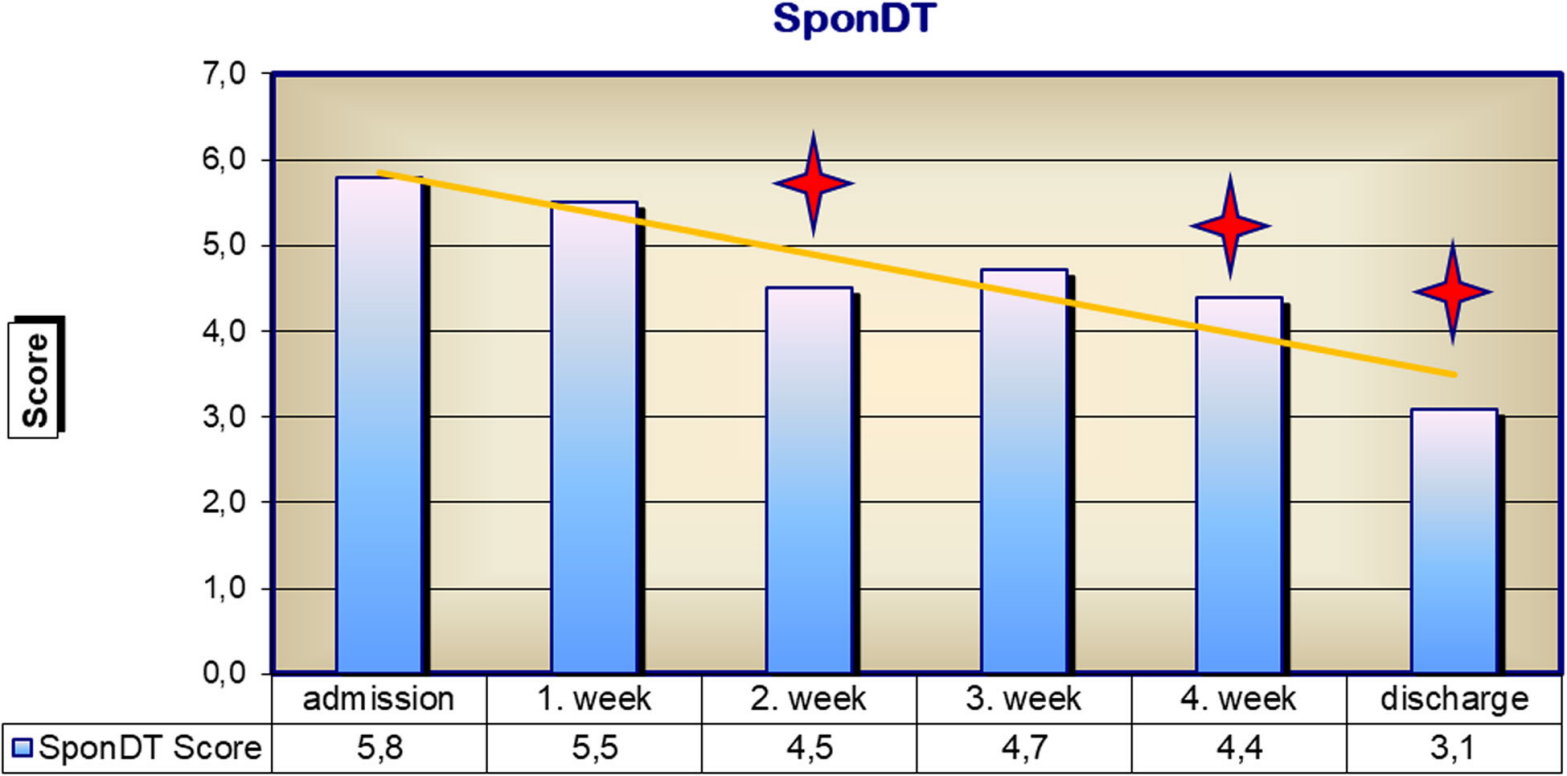
SponDT recording at admission to hospital and during the course of treatment; *p < 0.05

This classification of severity of spondylodiscitis was made on the basis of SponDT. It showed that 21% of patient’s initially had severe spondylodiscitis and 71.6% a moderate infection with spondylodiscitis. The SponDT score at admission was 5.6. Already in the second week of treatment, the proportion of severe cases of spondylodiscitis was down to 6.3%. The SponDT decreased to 4.4 in the second week and further up to 3.1 in the fourth week or at discharge (Fig. 6). According to that, cases of light spondylodiscitis infection or of healed patients increased from an initial 5.7 to 59.5%. A severe spondylodiscitis in the fourth week was less than 2%.

Spondylodiscitis is a rare illness with non-specific symptoms and is therefore likely to go misdiagnosed. We regularly treat around 20 cases per year. Yet it is also severe, and therefore timely and accurate diagnosis in the outpatient setting is already of immense importance for disease outcome [1, 3]. Our data support the view that spondylodiscitis is primarily a disease of old age (60–80-year age group) that more often afflicts men. We were able to show, contrary to the prevailing view, that surgical operations were the main cause of spondylodiscitis [1–3]. Spondylodiscitis was not only associated with spinal surgery but also with other forms of surgery, though other operations inevitably limited the possibilities to diagnose and treat spondylodiscitis, delaying the first diagnosis by 2 months after first symptoms [1, 3].

Because of the currently poor possibilities to diagnose spondylodiscitis, we set ourselves the goal to optimize treatment of the former. In addition to clinical examination, we propose the use of additional diagnostic parameters for SponDT, namely magnetic resonance imaging [12, 20] and pain assessment of patients with newly arisen back pain or for those whose back pain has lasted several days or that have a reduced general condition. We also recommend laboratory chemical tests of inflammation. These parameters can even be collected from outpatients and fed into SponDT.

MRI shows high sensitivity and specificity for the detection of spondylodiscitis, with over 90% of cases recognized [12, 20]. Although current data for MRI-based morphological classification of spondylodiscitis is very heterogeneous and not adapted to specific parameters associated with spondylodiscitis, MRI morphological analysis should nevertheless form part of the diagnosis of spondylodiscitis and evaluation of its treatment. Inflammatory markers are also typically elevated in cases of spondylodiscitis. The leukocyte count itself is rather non-specific. An increased CRP has, however, been found to be typical of spondylodiscitis, with a sensitivity of 84% and a specificity of 71% [4, 14]. For this reason, these parameters of SponDT are also used foremost in the treatment control of spondylodiscitis. The rather non-specific pain statement and reduction in general condition, as defined by the ASA classification, in contrast, have great importance in assessing the course of further treatment after initial diagnosis. Therefore, we proposed that the subjective perception of pain by the patient is a valid parameter for assessing the success of treatment [23].

Successful antibiotic treatment of spondylodiscitis decreases inflammation [3, 4, 26]. Our studies also show that there is a parallel reduction in the specific inflammatory parameter CRP following successful treatment. Absolute leukocyte count does not change, illustrating its lack of specificity for the diagnosis of spondylodiscitis [27]. Nevertheless, our studies show that the number of patients with a leukocyte count > 15 Gpt/l at 4 weeks post treatment was significantly reduced.

We therefore believe that a summary score like SponDT, generated by the parameters CRP, pain and MRI is a useful tool to help diagnose spondylodiscitis and to follow its outcome following treatment. In addition, SponDT allows the severity of spondylodiscitis to be classified.

On admission for spondylodiscitis and during the first week of treatment, our patients had an average SponDT score of 5.6 out of a maximum of 9 points. Of all of our patients, 20% had severe spondylodiscitis with scores > 6. This proportion decreased during the course of treatment to less than 2%, suggesting that the treatment was successful. However, the SponDT score of spondylodiscitis patients did not fall following treatment to the ideal value of 0 at discharge, due to lingering pain and persistent findings in the final radiological examination (MRI). We therefore suggest as the goal of spondylodiscitis treatment a SponDT score of less than 3 points. We note that this means that we cannot draw a distinction between complete remission and a light spondylodiscitis infection. Rather, the purpose of SponDT is to allow individual, patient-based, accurate documentation of the success of treatment. In this context, we see it as justified to incorporate the parameter of pain assessment into SponDT.

In the current literature, spondylodiscitis is associated with a mortality of 2–12% [3, 28, 29]. We registered a nearly constant rate of mortality of 7% in our patients, which illustrates the present risk of this disease and the current difficulty in its treatment.

In contrast, our studies suggest that MRI diagnosis cannot be used on its own in monitoring a course of treatment against spondylodiscitis. Though it is an essential component of spondylodiscitis diagnosis [3, 12, 20], it is of lesser value in following disease progression during treatment because it generates artefacts that hinder accurate assessment of disease severity. In addition, the rather specific features that MRI detects in spondylodiscitis patients to not change during the first 2–4 weeks of treatment, apart from possible surgically treated abscesses. Therefore, the main focus of magnetic resonance imaging within SponDT is in the initial assessment of the severity of the disease. Thereafter, during treatment of the disease, levels of inflammatory markers and pain intensity take a more important role in monitoring the progression of spondylodiscitis, independent of the age of the patient.

## Conclusion

In summary, our studies show that the combination of several diagnostic parameters into a single scoring system, SponDT, facilitates the diagnosis of spondylodiscitis. In combination with the spondylodiscitis severity code (SSC), a classification of the severity of spondylodiscitis, SponDT could be established and used for a severity-based treatment. In addition, specific parameters for the treatment of individual grades of severity can be determined in a clinical pathway [30]. Furthermore, the organizing and optimization of treatment remain in the scientific focus and form the basis of stage-appropriate spondylodiscitis therapy by means of classification [31, 32].

Likewise, in the field of surgical therapy, minimally invasive and gentle surgical procedures are the focus of scientific consideration. The minimally invasive thoracic approach of the spine and the implantation of PEEK or titanium cages avoid the use of bone graft [33, 34].

MRI and laboratory inflammatory markers are essential to this scoring system. While the latter retain their diagnostic importance in monitoring the course of treatment of spinal osteomyelitis, the use of MRI is not justified in cases of spondylodiscitis arising from surgical treatment of the spinal region; rather, clinicians should resort to the use of CT for monitoring the progression of spondylodiscitis. We stress that subjective pain perception is an integral and important parameter of SponDT for treatment evaluation. SponDT can be used in both the initial diagnosis of spondylodiscitis and in disease progression during treatment.

## Abbreviations

CRP: C-reactive protein
NRS: Numeric rating scale
MRI: Magnetic resonance imaging
CT: Computer tomography
ASA: American Society of Anesthesiologists
NIDDM: Non-insulin dependent diabetes mellitus
IDDM: Insulin dependent diabetes mellitus
SSC: Spondylodiscitis severity code
SponDT: Spondylodiscitis Diagnosis and Treatment
